# Supplementary material for: Understanding health system responsiveness to public feedback at the sub-national level: Insights from Kilifi County, Kenya
Source: PLOS Glob Public Health. 2024 Dec 12;4(12):e0002814. doi: 10.1371/journal.pgph.0002814 (PMC11637338; doi:10.1371/journal.pgph.0002814)
Supplement: S2 File — (DOCX) [file pgph.0002814.s003.docx]

**S2 File: Observation Checklist for SCHMT meetings and activities**

***Provide description of:***

| **Structuring observations (Adapted from Ann Bowling(Bowling, 2014)** | |
| --- | --- |
| 1. **Setting** | What is the physical environment like? How is the SCHMT room organised? Physical objects related to broad county health policy-E.g. posters, mission, vision statement of the County Department of Health, any posters/notices related to policy around receiving and responding to public feedback? |
| 1. **Participants** | Who are the members of the SCHMT? How many people and their roles? |
| 1. **Activities and interactions** | Who initiates the meeting? Who speaks most frequently? Are there interruptions to when a team member talking? How are these responded to by the one who is speaking? By other team members? Do all SCHMT members actively participate in discussions? Are there members who are quiet during most of the meetings? Are the meetings documented? Who is responsible for documentation (One specific person/rotational? Is there a structure to the documentation?  Does there seem to be a ‘core’ SCHMT team? What makes them ‘core’ (e.g. control over/ access to resources, professional back-ground etc). If present, how does this core team interact with the rest of the team? (E.g. are they inclusive in decision-making about SCHMT activities) |
| 1. **Content of discussion** | Is there a standing agenda? Is public feedback among the items on the standing agenda? If not, how frequently has public feedback come up in discussions? When public feedback was discussed, how much time was spent on it, were there specific, actionable resolutions?  -Are there sub-committees within the SCHMT that discuss public feedback? Which members of the SCHMT are involved in this sub-committee? How were they selected? |
| 1. **Frequency and duration** | How frequently do the SCHMT meetings occur? How long on average do they last? When SCHMT meetings are not held, what are the reasons for not holding them? |
| 1. **Other activities outside of meetings** | What other activities are carried out apart from support supervision activities and vaccine campaigns? How frequent if any are unplanned activities? What is the reaction to these unplanned activities? |
| 1. **(Common Language)** | Are symbolic and connotative meanings of words; non-verbal communication (e.g. dress, space)Are there any negative labels or identities assigned to other actors (e.g. patients, community members, general public, senior health managers, political representatives) or physical spaces (e.g. specific health centres, county health managers’ offices) Instances of humour camouflaged as dissent/disagreement? |
