# Supplementary material for: Understanding health system responsiveness to public feedback at the sub-national level: Insights from Kilifi County, Kenya
Source: PLOS Glob Public Health. 2024 Dec 12;4(12):e0002814. doi: 10.1371/journal.pgph.0002814 (PMC11637338; doi:10.1371/journal.pgph.0002814)
Supplement: Attachment — Submitted filename: Cover letter and Response to Reviewers.pdf [file pgph.0002814.s004.pdf]

Nancy Kagwanja
KEMRI-Wellcome Trust,
P.O Box 230-80108, Kilifi, Kenya
Nkagwanja@kemri-wellcome.org
23/08/2024

Carl Abelardo T. Antonio
Academic Editor
PLOS Global Public Health

Dear Carl Abelardo,

RE: Resubmission: Understanding health systems responsiveness to public feedback at the sub-national level in Kenya

We thank both reviewers for their comments. We have also included the checklist for inclusivity in global health research as supporting information.

Please see below, point by point responses to the comments raised by the reviewers.

General comment: This is an interesting and important topic but needs elaboration.

Specific comments

1. The title can be misleading since the study used a case study in Kilifi County, instead of Kenya. Hence, Kilifi County should be in the title.

We have now included Kilifi County in the title

2. Abstract contains some local-context terminologies e.g. Health Facility committees and sub-county health management teams, which are hard to follow. The abstract should be rewritten.

We have re-written a section of the abstract to explain this terms, (pg 2, lines 38-44).

3. Materials and methods

- Study setting: The authors should explain the connections between institutions stated in the study, such as Health facilities committed, sub-county health management systems dan county, and members of the county assembly as well as the responsibility between the institutions in the health systems.

We have included a figure that shows the connections between HFCs, the Sub-county health management team and other actors within the county health system, and external to it (Fig 1, pg 5).

- The selection of Kilifi County should be more explained instead of the convenience of the researcher due to existing collaboration. The sampling methods should consider relevance and urgency.

We have re-framed our justification for Kilifi County as a study site. Kilifi County was a good fit for the study because of the embeddedness of health policy and system researchers, an approach which supports understanding of context, translation, and uptake of research findings (pg 5, lines 109-115).

- The author should cite references on the qualitative case study that was used.

We have now included references to the case study approach (pg 6, lines 138-139)

- Data collection: the authors should explain the content of guidelines for in-depth interviews, focus group discussion, and observation.

The interview and FGD topic guides included questions about the nature of feedback received by the HFCs and SCHMTs, what channels members of the public utilised to provide feedback, whether and what responses were generated to this feedback (pg 7, lines 158-161; pg 7-8 lines 162-165). The topic and observation guides have been included in the Supporting information 1 and 2.

4. Results

- Box 1 needs for elaboration. What are the definitions of participatory, bidirectional, and unidirectional mechanisms?

We have provided definitions of terms used in Box 1 as follows on pg 9, lines 194-199: 'participatory mechanisms are those in which the public are invited to contribute feedback, and there is opportunity for bi-directional or two-way engagement between the public and health system actors. Uni-directional mechanisms support the public to share feedback with health system actors but mainly involve collection of feedback, thus feedback flows in one direction and there is little engagement at the point of providing feedback'.

- Table 3 needs for explanation in the text.

We have provided some in-text explanation for Table 3 describing that Table 3 summarises functionality details about feedback mechanisms and highlights various system hardware and software constraints that cut across both the uni-directional and participatory mechanisms. We have provided a summary of these hardware and software constraints (pg 10; lines 205-209). We have also made additional references to Table 3 (pg 11, line 218; pg 12, lines 232-233 to link back the data presented in Table 3 to the subsequent sections of text.

- Fig 3 needs for explanation in the text.

-We have provided a description of the overall nature of issues raised by members of the public depicted in Fig. 4 (previously Fig 3), and highlighted the broad categories of feedback as cutting across four broad areas (healthcare worker conduct; service delivery processes, commodity and infrastructure requests, and resistance to public health initiatives (pg , and for each area we have highlighted some examples. experiences of healthcare worker conduct, requests for changes in service delivery processes, commodity and infrastructure requests, and resistance to participation in public health initiatives (pg 16, lines 69-74)

5. Discussion
- Need to add implications for low-and middle-income countries.

We have reframed the second-last and last paragraphs in the discussion section to present the implications of weak health system responsiveness for LMICs, which include a lack of trust in the health system that could undermine willingness amongst members of the public to provide input, and to comply with health system or public health initiatives (pg 24, lines 292-294; 297-303).

- Study limitations of the case study should be elaborated

We have included study limitations of the case study approach. These are mainly concerns about generalisability of case study findings, but we have highlighted that the study findings would still be transferable to similar contexts, such as other Low-and-Middle-Income Countries (pg 29, lines 320-325)

Reviewer #2: This paper offers an important and necessary exploration of the challenges of health system feedback systems in Kenya. It surfaces really important soft and hard barriers to better feedback systems. The way the study highlights informal and formal mechanisms is important and quite novel.

Most of my concerns with the paper are around the rigor and appropriateness of the theoretical frames and analysis. The primary analysis focuses on domains of organizational capacity, using a software / hardware terminology that is especially confusing. Perhaps instead use intangible / tangible? Or even the use of hard vs. soft capacities would be easier to understand.
*note there is no reference provided for Aragon (which should be listed as Ortiz Aragon?). In addition, I think the Ortiz Aragon piece you are referring to, "Ortiz Aragón, A. A Case for Surfacing Theories of Change for Purposeful Organisational Capacity Development," actually does not use the terms software or hardware either. In addition, the framework from Aragon that is being used here is not adequately explained in order to allow readers who are not familiar with it to understand your usage.
We have now included a reference for the paper by Ortiz Aragon, and rephrased our wording to show that these ideas (about system software and hardware) are drawn originally from Aragon's paper, but have also been used in other papers on health policy and systems research. We acknowledge that this paper (A Case for Surfacing Theories of Change for Purposeful Organisational Capacity Development) uses the terms 'hard' and 'soft' rather than 'software and hardware' but other health policy and system researchers who make the distinction between these elements use the latter terms, and so we have retained their use in this paper (see: Elloker, S., Olckers, P., Gilson, L., & Lehmann, U. (2012). Crises, routines and innovations: the complexities and possibilities of sub-district management. South African health review, 2012(1), 161-173

Sheikh K, Gilson L, Agyepong IA, Hanson K, Ssengooba F, Bennett S. Building the field of health policy and systems research: framing the questions. PLoS Med. 2011 Aug;8(8):e1001073. doi: 10.1371/journal.pmed.1001073.

See rewording on pg 8, lines 174-182 where we have provided more detail on the hardware/software components of the health system and how we have used them in this work.

It seems that perhaps using a power analysis might be a more helpful framework through which to analyze some of these results. It's mentioned fairly briefly on p. 24 but a more extensive power analysis throughout might be helpful. Simply categorizing power as an "intangible software element" does not do it justice, I think.

Thank you for this feedback. We did not include a power analysis in this paper because we first wanted to describe the practice of responsiveness (including what responses are generated within the health system), to make the case that responsiveness is complex, and multi-faceted, before considering how power impacts responsiveness. We think that there is value in the approach we have taken of applying a broad systems lens, and distinguishing hardware and software dimensions given that much of the responsiveness literature focuses on service-provider interactions, as has been identified in two recent reviews of the responsiveness literature:

i. *Khan G, Kagwanja N, Whyle E, Gilson L, Molyneux S, Schaay N, Tsofa B, Barasa E, Olivier J. Health system responsiveness: a systematic evidence mapping review of the global literature. Int J Equity Health. 2021 May 1;20(1):112. doi: 10.1186/s12939-021-01447-w.*
ii. *Mirzoev T, Kane S. What is health systems responsiveness? Review of existing knowledge and proposed conceptual framework. BMJ Glob Health. 2017 Oct 31;2(4):e000486. doi: 10.1136/bmjgh-2017-000486)*

We agree that an extensive power analysis is important, and we have done this in a companion paper that has been submitted elsewhere, and is currently under review (pg 27, lines 281-285)

Fig 1's conceptual framework does not really include adequate information on how feedback is solicited / offered from patients / marginalized groups. Perhaps consider integrating some of the flows represented in Box 1 into the Fig. 1.

We have made changes to Fig 1 (this is now Fig. 2) to include different channels through which the 'processing space' could receive input from the public that is either formal (participatory or uni-directional) and informal channels

Theoretically, it might also be useful to explore some of the longer histories (and failures) of participation initiatives in African states, such as those by Cooke and Kothcari (2001), which described participatory approaches as a "new tyrrany." The paper also reminded me of earlier histories of efforts to decentralize and democratize HIV program implementations, such as Nora Kenworthy's Mistreated (2017) (which discusses at length local institutions very similar to the HCFs). To this end, it might be helpful to think about why similar structures for participation and feedback keep getting (re)created in different waves of global health implementation over decades, but with similarly dismal results when it comes to improving citizen participation in health systems.

We have included in the last paragraph of the discussion some ways that participation and feedback can be re-imagined and suggested that a conceptualisation of participation would need to include

consideration of broader contexts such as the social, political and economic spheres that these mechanisms act. We also suggest that the outcomes of public participation and feedback could be informed by drawing on theories from the literature on power, participatory approaches and political science (Pg 28-29, lines 305-317)

While exclusion of marginalized groups is highlighted in the paper abstract and introduction as a major focus of the piece, it receives very limited space in the actual results section (mostly on p. 20-21). I would suggest giving this some greater attention in the discussion of results if you intend to keep it as a major focus of the findings. Additionally, thinking about how power / hierarchy / stigma play here is important.

We have now included some additional findings about generation of responses to vulnerable and/or marginalised groups in the results section (pg 24, lines 196-204). We have also included in the discussion section, a paragraph about the experiences of vulnerable groups, how it reflects on equity in responsiveness, and have linked it to power dynamics (pg 27, lines 274-285). Though we have suggested that structural forms of power could be linked to the exclusion of vulnerable groups, we have not done an in-depth exploration of the forms of power at play as we felt that such an analysis would be more effectively addressed in another paper. We have done this in a paper focused specifically on the influence of power on responsiveness practices which is currently under review. The pre-print details are:

Kagwanja, N., Molyneux, S., Whyle, E., Tsofa, B., Leli, H., & Gilson, L. (2024). Power and positionality in the practice of health system responsiveness at sub-national level: insights from the Kenyan Coast, available at http://dx.doi.org/10.21203/rs.3.rs-4430438/v1

Sincerely,

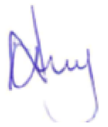

Nancy Kagwanja
